# Protective Effects of Adiponectin against Cobalt Chloride-Induced Apoptosis of Smooth Muscle Cells via cAMP/PKA Pathway

**DOI:** 10.1155/2020/7169348

**Published:** 2020-10-10

**Authors:** Jingjie Xiao, Yingying Zhang, Wei Zhang, Liang Zhang, Li Li, Junqiang Si, Xinzhi Li, Ketao Ma

**Affiliations:** ^1^Department of Physiology, Medicine School of Shihezi University, Shihezi, Xinjiang, China; ^2^Department of Biochemistry, Wuhan University School of Basic Medical Sciences, Wuhan, China; ^3^Key Laboratory of Xinjiang Endemic and Ethnic Diseases, Medicine School of Shihezi University, Shihezi, Xinjiang, China; ^4^Department of Gastroenterology, People's Hospital of Jiaozuo city, Jiaozuo, China; ^5^Department of Pathophysiology, Medicine School of Shihezi University, Shihezi, Xinjiang, China

## Abstract

Adiponectin (APN) is an adipokine secreted from adipose tissue and exhibits biological functions such as microcirculation-regulating, hearing-protective, and antiapoptotic. However, the effect of APN on the apoptosis of spiral arterial smooth muscle cells (SMCs) under hypoxic conditions in vitro is not clear. We used cobalt chloride (CoCl_2_) to simulate chemical hypoxia in vitro, and the SMCs were pretreated with APN and then stimulated with CoCl_2_. The viability of cells and apoptosis were assessed by CCK-8 and flow cytometry, respectively. Superoxide dismutase (SOD) activity, malondialdehyde (MDA) levels, cAMP level, and the activity of PKA were detected by ELISA. Protein expression and localization were studied by Western blot and immunofluorescence analysis. In the present study, we found that APN exhibits antiapoptosis effects. CoCl_2_ exhibited decreased cell viability, increased apoptosis and MDA levels, and decreased SOD activity in a concentration-dependent manner, compared with the control group. Moreover, CoCl_2_ upregulated the expression levels of Bax and cleaved caspase-3 and then downregulated Bcl-2 levels in a time-dependent manner. Compared with the CoCl_2_ group, the group pretreated with APN had increased cell viability, SOD activity, PKA activity, cAMP level, and PKA expression, but decreased MDA levels and apoptosis. Lastly, the protective effect of APN was blocked by cAMP inhibitor SQ22536 and PKA inhibitor H 89. These results showed that APN protected SMCs against CoCl_2_-induced hypoxic injury via the cAMP/PKA signaling pathway.

## 1. Introduction

Numerous studies have shown that cochlear function is sensitive to dynamic changes in cochlear blood flow, and disorders of the inner ear circulatory system have caused diseases to plague a large number of patients worldwide, such as noise-induced and sudden sensorineural hearing loss. Furthermore, regulation of cochlear blood flow is essential for hearing and is important as a treatment strategy for the restoration of hearing loss in humans [1, 2]. In addition, due to the high energy consumption of auditory conduction, the blood supply of the spiral modiolar artery (SMA) is considered essential for maintaining the function of the hearing device [3]. And studies on the inner ear circulatory system have confirmed that, on the one hand, the cochlear strong ability of blood flow autonomous regulation could ensure the blood supply of local tissues, and on the other hand, this ability could reduce the impact of the rapid changes of blood pressure and also maintain water and electrolyte balance in the local tissues of the cochlea [4].

The SMA is the primary (or main) artery responsible for supplying blood to the cochlea, and its upstream arteries are the anterior inferior cerebellar artery and the basilar artery. The anterior inferior cerebellar artery has two functional terminal branches, namely, the vestibular cochlear and spiral artery branches [5]. Hence, the cochlea is highly dependent on blood and oxygen supply. Abnormal changes in the SMA, such as vasospasm, could reduce or even completely interrupt cochlear blood supply [6]. In other words, sufficient blood flow in SMA is a necessary condition for maintaining normal hearing. In addition, cobalt chloride (CoCl_2_) is a hypoxia-mimicking agent that is commonly used in hypoxic culture studies. CoCl_2_ mimics the hypoxic response by inhibiting the activity of prolyl hydroxylase, a key enzyme in the oxygen sensing pathway [7]. These abnormal conditions lead to blood circulation disorders in the inner ear and cochlear dysfunction, which may result in dizziness, tinnitus, and other symptoms [8].

The cAMP/protein kinase A (PKA) signal transduction pathway is a classical cellular signaling pathway mediated by G protein-coupled receptors, which are involved in the regulation of cell differentiation, proliferation, apoptosis, and gene transcription. Studies have shown that PKA not only plays an important role in apoptosis, but also cAMP/PKA pathway is involved in the hypoxia-induced suppression of the genes [9]. So, our goal was to determine if a known antioxidant and antiapoptotic chemical would reduce hypoxic effects through the cAMP/PKA pathway.

Adiponectin (APN) is an adipokine secreted from adipose tissue and plasma with concentrations ranging from 3 to 30 *μ*g/ml in mouse and human. It has anti-inflammatory, antiapoptosis, antiatherosclerosis, and microcirculatory functions through complex and diverse signal transduction pathways, and it promotes the utilisation of glucose and oxidation of fatty acids, lowers blood sugar, and improves insulin resistance [10, 11]. Based on the above research status, we speculate that APN may exhibit antiapoptosis effects on smooth muscle cells (SMCs) of SMA not only by regulating the levels of superoxide dismutase (SOD) and malondialdehyde (MDA) but also by regulating the expression levels of Bax, Bcl-2, and cleaved caspase-3 through the cAMP/PKA signaling pathway.

## 2. Material and Methods

### 2.1. Cell Culture and Experiment Design

Highly purified SMCs in passage 4 (P4) were obtained from the SMA of guinea pigs and used for all experiments, as described previously [12]. The SMCs were cultured with 10% foetal bovine serum (FBS) (Gibco, Carlsbad, CA, USA), 100 units/ml penicillin, and 100 *μ*g/ml streptomycin in an incubator containing 5% CO_2_ at 37°C. The cells were cultured in 25 cm^2^ culture flasks, and the medium was changed every 2-3 days. Upon reaching 80%-90% confluence, the cells were passaged. Then, the SMCs were treated with CoCl_2_ (#C8661, Sigma-Aldrich, USA) of 0, 25, 50, 100, 200, and 400 *μ*M for 0, 3, 6, 12, 24, and 48 h [13, 14]. Cells were pretreated with APN (#0911545, PeproTech, Rocky, USA, 2 *μ*g/ml, 2 h), followed by CoCl_2_ (100 *μ*M, 24 h). To elucidate the role of the cAMP/PKA signaling pathway, the cells were pretreated with cAMP inhibitor SQ22536 (#S8283, Selleck Chemicals, USA, 1 mM, 30 min) or PKA inhibitor H 89 (#S1582, Selleck Chemicals, USA, 50 *μ*M, 1 h) before being treated with CoCl_2_ and APN.

### 2.2. Cell Counting Kit-8 (CCK-8) Assay

The viability of cells was measured using a CCK-8 (MultiSciences Lianke Biotech Co., Ltd. China) following the manufacturer's instructions. Briefly, 5 × 10^3^ cells per well were plated in 96-well plates and incubated in 37°C. After designated treatments, 10 *μ*l CCK-8 was added in each well, and the 96-well plate was incubated in 37°C for 2 h. Absorbance at 450 nm wavelength of each well was determined by using a microplate reader (Bio-Rad, Hercules, CA, USA).

### 2.3. Immunofluorescence Analysis

After designed treatment, the cells were fixed in 4% paraformaldehyde for 15 min, permeabilised with 0.2% Triton X-100 for 3 min and incubated with 5% BSA for 30 min, and primary antibodies were added at 4°C overnight. Antibodies to PKA (1 : 100; No. ab38949), Bax (1 : 200; No. ab199677), Bcl-2 (1 : 100; No. ab196495), and caspase-3 (1 : 200; No. ab13847) were obtained from Abcam. The next day, the cells were rewarmed for 30 min at 37°C, and secondary antibodies were added in a dark room 37°C for 1 h, followed by incubation with DAPI out of light for 5 minutes. A confocal microscopy (#510, Zeiss LSM, Germany) was used to view the results.

### 2.4. ELISA

The SOD and MDA enzyme immunoassay assay kit (Jiancheng Bioengineering Institute, Nanjing, China) were used to determine SOD activity and MDA levels according to the manufacturer's instructions. Also, the cAMP enzyme immunoassay assay kit and PKA activity assay kit (Solarbio Science and Technology Co., Beijing, China) were used to determine cAMP level and the activity of PKA according to the manufacturer's instructions.

### 2.5. Cell Apoptosis Analysis

Cell apoptosis was detected by propidium iodide (PI) and Annexin V-FITC staining according to the manufacturer's instructions (MultiSciences Lianke Biotech Co., Ltd. Hangzhou, China). Briefly, after designed treatment, cells were harvested and stained with PI and Annexin V-FITC, and cells were cultured without light at room temperature. 15 minutes later, each reaction tube was added with 400 *μ*l of binding buffer. Apoptosis of cells was analyzed by a FACSAria™ flow cytometer (BD Biosciences, USA), and the data were analyzed using the FlowJo 7.6 software (FlowJo, LLC, Ashland, USA).

### 2.6. Western Blot Analysis

After designed treatment, whole cell extracts were collected and lysed with lysis buffer. The extracted protein samples (about 40 *μ*g) were separated by SDS-PAGE (10–15%) and transferred onto PVDF membranes. After being blocked with 5% nonfat milk for 2 h, the membranes were incubated with primary antibodies. Antibodies to PKA (1 : 1,000; No. ab38949), Bax (1 : 1,000; No. ab199677), Bcl-2 (1 : 500; No. ab196495), and caspase-3 (1 : 500; No. ab13847) were obtained from Abcam. After that, the membranes were washed and incubated with secondary antibodies conjugated with horseradish peroxidase (HRP) for 1-2 h at room temperature. Next, GAPDH or *β*-actin served as a loading control. Finally, the signals were analyzed using a chemiluminescence (ECL) detection kit. The ImageJ software was used for semiquantitative calculations.

### 2.7. Statistical Analysis

All values are presented as the mean ± SE. Statistical data were analyzed using SPSS 22.0 (IBM Corp., Armonk, NY, USA). Unpaired, two-tailed Student's *t*-test was used for two groups. One-way analysis of variance (one-way ANOVA) with Tukey's post hoc test was performed to compare multiple groups. GraphPad Prism (v.7.0; GraphPad Software Inc., San Diego, CA, USA) was used to analyze data and prepare all figures. *P* values less than 0.05 (*P* < 0.05) indicated significant differences.

## 3. Results

### 3.1. CoCl_2_ Reduced SMCs Viability and Increased Apoptosis in a Concentration-Dependent Manner

**Figure 1:**
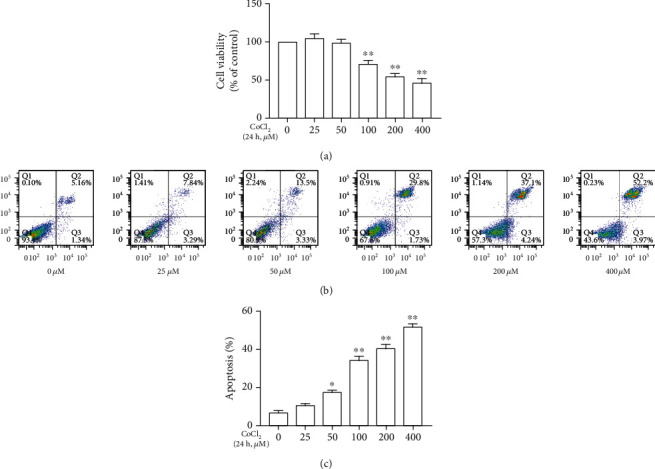
CoCl_2_ reduced SMCs viability and increased apoptosis in a concentration-dependent manner. (a) CoCl_2_ of 0, 25, 50, 100, 200, and 400 *μ*M treatment for 24 h reduced the viability of SMCs in a concentration-dependent manner. (b) CoCl_2_ of 0, 25, 50, 100, 200, and 400 treatment for 24 h increased apoptosis in a concentration-dependent manner. (c) Statistical analysis of the rate of apoptosis in each group. The viability of SMCs were measured by CCK-8 and the rate of apoptosis were measured by flow cytometry (∗*P* < 0.05 vs. control, ∗∗*P* < 0.01 vs. control, *n* = 5, data shown as the mean ± SE). CoCl_2_: cobalt chloride.

As shown in Figures 1(a)–1(c), CoCl_2_ of 0, 25, 50, 100, 200, and 400 *μ*M treatment for 24 h induced cell injury and increased apoptosis in a concentration-dependent manner. Among them, CoCl_2_ of 100, 200, and 400 *μ*M has a significant effect on cells.

### 3.2. CoCl_2_ Affects Apoptotic Markers in a Concentration- and Time-Dependent Manner

**Figure 2:**
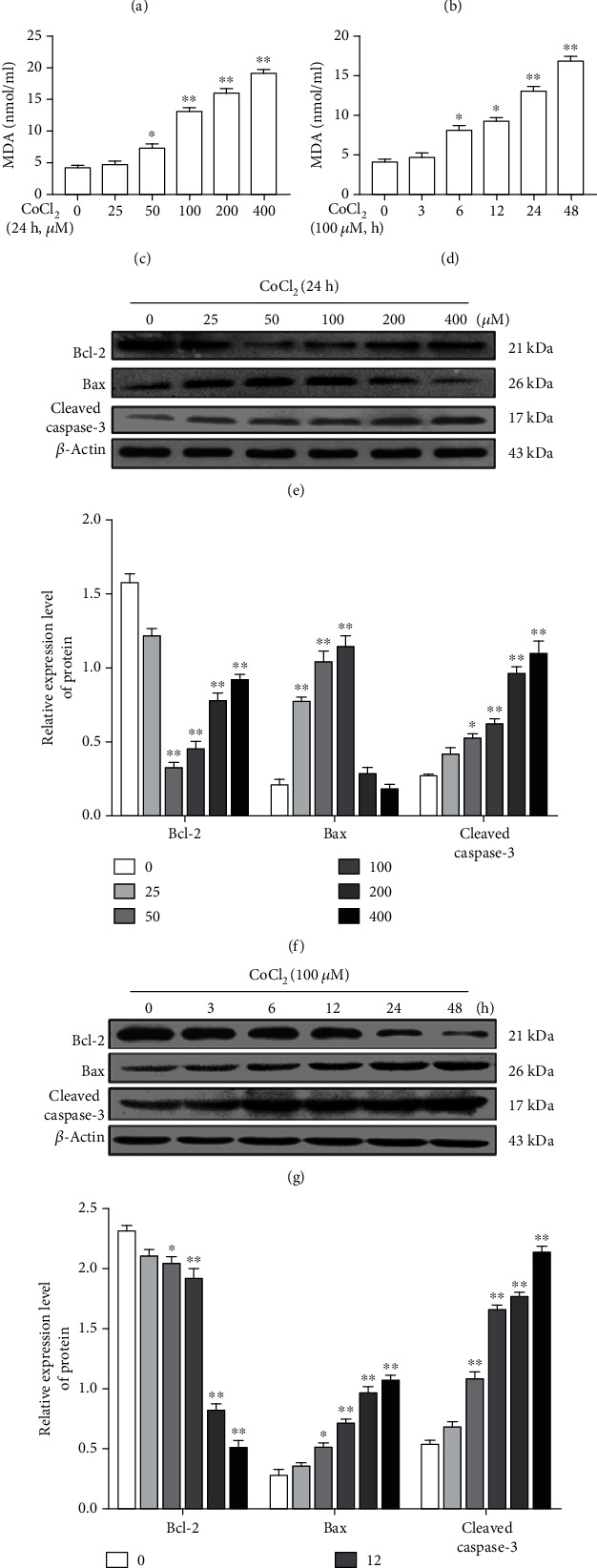
CoCl_2_ affects apoptotic markers in a concentration- and time-dependent manner. (a) CoCl_2_ reduced SOD activity in a concentration-dependent manner. (b) CoCl_2_ reduced SOD activity in a time-dependent manner. (c) CoCl_2_ increased MDA levels in a concentration-dependent manner. (d) CoCl_2_ increased MDA levels in a time-dependent manner. (e) CoCl_2_ (50, 100, and 200 *μ*M) significantly upregulated Bax and cleaved caspase-3 expression and downregulated Bcl-2 expression in SMCs. (f) Statistical analysis of the expression of Bcl-2, Bax, and cleaved caspase-3. (g) CoCl_2_ (100 *μ*M) upregulated Bax and cleaved caspase-3 expression and downregulated Bcl-2 expression in a time-dependent manner in SMCs. (h) Statistical analysis of the expression of Bcl-2, Bax, and cleaved caspase-3 (∗*P* < 0.05 vs. control, ∗∗*P* < 0.01 vs. control, *n* = 5, data shown as the mean ± SE). CoCl_2_: cobalt chloride; SOD: superoxide dismutase; MDA: malondialdehyde; Bcl-2: B-cell lymphoma 2; Bax: Bcl-2-associated X protein.

Compared with the control group, as shown in Figure 2(a), cells treated with CoCl_2_ (100, 200, and 400 *μ*M) obviously reduced SOD activity, and 100 *μ*M CoCl_2_ treatment for 24 and 48 h has significant effects on it (Figure 2(b)). Moreover, CoCl_2_ of 100, 200, and 400 *μ*M treatment for 24 h significantly increased MDA levels (Figure 2(c)), and as shown in Figure 2(d), 100 *μ*M CoCl_2_ treatment for 24 and 48 h also significantly increased MDA levels. In addition to the effects of CoCl_2_ on SOD activity and MDA levels, it can also affect the expression of apoptotic-related proteins. Specifically, CoCl_2_ of 50, 100, and 200 *μ*M treatment for 24 h upregulated the expression levels of Bax and cleaved caspase-3 and downregulated those of Bcl-2. It was worth noting that 400 *μ*M CoCl_2_ had no significant effect on the expression of Bax and Bcl-2 but significantly upregulated the expression levels of cleaved caspase-3 (Figures 2(e) and 2(f)). As shown in Figures 2(g) and 2(h), 100 *μ*M CoCl_2_ upregulated the expression levels of Bax and cleaved caspase-3 and downregulated those of Bcl-2 in a time-dependent manner.

### 3.3. APN Protects against the Apoptotic Responses of Cells to CoCl_2_

**Figure 3:**
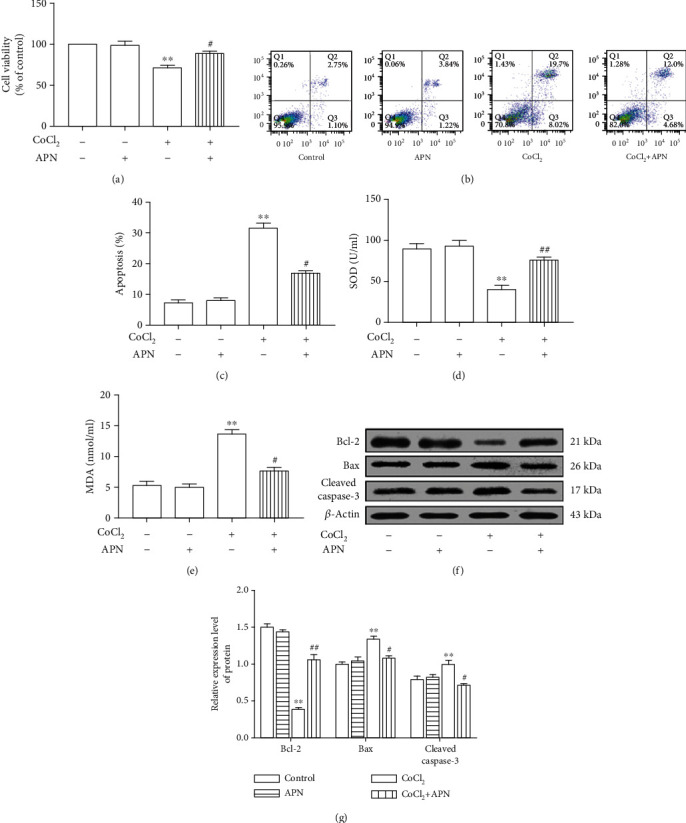
APN protects against the apoptotic responses of cells to CoCl_2_. (a) The CoCl_2_-induced reduction SMCs viability was attenuated by APN. (b) Statistical analysis of the rate of apoptosis in each group. (c) The CoCl_2_-induced increase apoptosis was attenuated by APN. (d–f) The CoCl_2_-induced change of SOD activity, MDA levels, and Bax, Bcl-2, and cleaved caspase-3 expression all were blocked by APN. (g) Statistical analysis of the expression of Bcl-2, Bax, and cleaved caspase-3. (∗∗*P* < 0.01 vs. control, ^#^*P* < 0.05 vs. CoCl_2_ treatment, ^##^*P* < 0.01 vs. CoCl_2_ treatment, *n* = 5, data shown as the mean ± SE). CoCl_2_: cobalt chloride; APN: adiponectin; SOD: superoxide dismutase; MDA: malondialdehyde; Bcl-2: B-cell lymphoma 2; Bax: Bcl-2-associated X protein.

On the one hand, the SMC viability and activity of SOD were significantly decreased (Figures 3(a) and 3(d)), and the apoptosis and MDA levels in SMCs were significantly increased in the CoCl_2_-treated model group (Figures 3(b), 3(c), and 3(e)); on the other hand, CoCl_2_ upregulated the expression levels of Bax and cleaved caspase-3 and downregulated those of Bcl-2 (Figures 3(f) and 3(g)). However, these effects were blocked by APN. Moreover, compared with the control, the groups treated with APN alone had no differences in SMCs viability, SOD activity, MDA levels, apoptosis, or the expression of Bcl-2, Bax, and cleaved caspase-3 (Figures 3(a)–3(g)).

### 3.4. APN Protected against the CoCl_2_ Reduction of cAMP and PKA

**Figure 4:**
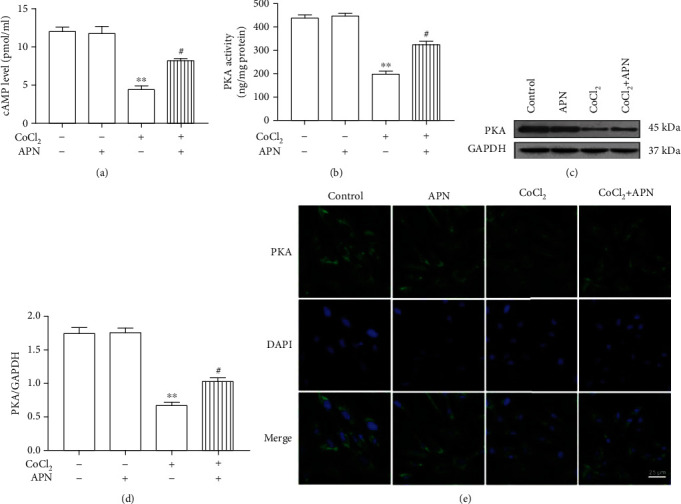
APN protected against the CoCl_2_ reduction of cAMP and PKA. (a) CoCl_2_-induced reduction of the cAMP levels was attenuated by APN. (b) CoCl_2_-induced reduction of the PKA activity was attenuated by APN. (c) PKA downregulation by CoCl_2_ was blocked by APN. (d) Statistical analysis of the PKA expression. (e) Expression and location of PKA (green) in the SMCs. Blue indicates nuclei as stained by DAPI. Scale bar: 25 *μ*m (∗∗*P* < 0.01 vs. control, ^#^*P* < 0.05 vs. CoCl_2_ treatment, *n* = 5, data shown as the mean ± SE). CoCl_2_: cobalt chloride; APN: adiponectin; cAMP: cyclic adenosine monophosphate; PKA: protein kinase A.

As shown in Figures 4(a) and 4(b), compared with control, CoCl_2_ (100 *μ*M, 24 h) decreased the level of cAMP and the activity of PKA and downregulated the expression levels of PKA (Figures 4(c) and 4(d)), but these effects were blocked by APN (2 *μ*g/ml, 2 h). Also, compared with the control, the groups treated with APN alone had no differences in the level of cAMP and the activity of PKA or the expression of PKA (Figures 4(a)–4(d)). Moreover, as shown in Figure 4(e), PKA was mainly distributed in the cytoplasm in SMCs.

### 3.5. cAMP and PKA Inhibition Blocked APN-Mediated Protection against CoCl_2_ Effects

**Figure 5:**
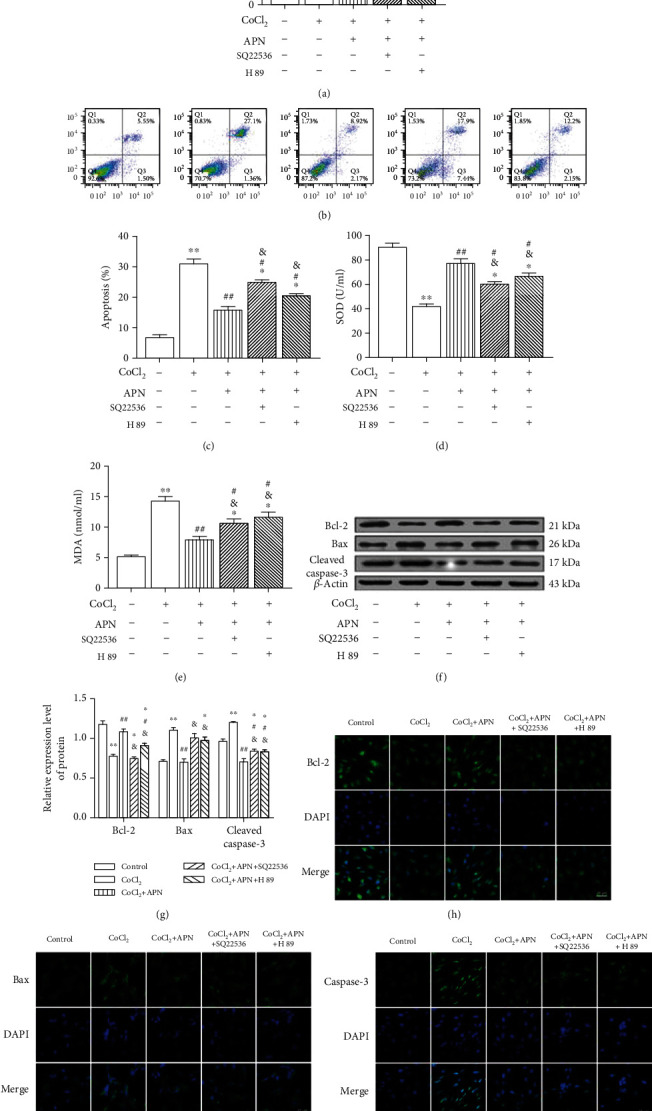
cAMP and PKA inhibition blocked APN-mediated protection against CoCl_2_ effects. (a, c–e) The CoCl_2_-induced reduction SMCs viability and SOD activity and increase apoptosis and MDA levels were blocked by APN; those effects of APN could be blocked by SQ22536 and H 89. (b) Statistical analysis of the rate of apoptosis in each group. (f) The CoCl_2_-induced Bax and cleaved caspase-3 upregulation and Bcl-2 downregulation were blocked by APN, and effects of APN could also be blocked by SQ22536 and H 89. (g) Statistical analysis of the expression of Bcl-2, Bax, and cleaved caspase-3. (h–j) Expression and location of Bcl-2, Bax, and caspase-3 (green) in the SMCs. Blue indicates nuclei as stained by DAPI. Scale bar: 25 *μ*m (∗*P* < 0.05 vs. control, ∗∗*P* < 0.01 vs. control, ^#^*P* < 0.05 vs. CoCl_2_, ^##^*P* < 0.01 vs. CoCl_2_, ^&^*P* < 0.05 vs. APN+CoCl_2_, *n* = 5, data shown as the mean ± SE). CoCl_2_: cobalt chloride; APN: adiponectin; SQ22536: cAMP inhibitor; H 89: PKA inhibitor; SOD: superoxide dismutase; MDA: malondialdehyde; Bcl-2: B-cell lymphoma 2; Bax: Bcl-2-associated X protein.

SMCs were pretreated with the cAMP inhibitor SQ22536 (1 mM, 30 min), the PKA inhibitor H 89 (50 *μ*M, 1 h), and APN (2 *μ*g/ml, 2 h) separately in the following experiment. As shown in Figures 5(a)–5(g), APN modulated the CoCl_2_-induced changes in SMCs viability, activity of SOD, MDA levels, rate of apoptosis, the expression levels of Bax, Bcl-2, and cleaved caspase-3, and the effects of APN were protected against by SQ22536 and H 89. Moreover, as shown in Figures 5(h)–5(j), Bax was mainly distributed in the cytoplasm, while Bcl-2 and caspase-3 were mainly distributed in the nucleus.

## 4. Discussion

The SMA is the main artery that supplies blood to the cochlea. Moreover, the SMA, a coiled artery inside the cochlea, is a branch of the anterior inferior cerebellar artery, which in turn branches off from the basilar artery located on the surface of the brain stem [5]. Given that the SMA is the only artery that supplies blood flow to the cochlea with low collateral circulation, once vasospasm or hypoxia occur, the blood flow will not be easy to compensate, and this could result in disturbances and pathological damage to the cochlear microcirculation [15]. Therefore, the cochlea is highly dependent on blood and oxygen supply. The labyrinthine artery is the terminal artery for cochlear blood supply, and interruption of this supply secondary to arterial damage causes immediate loss of function and inner ear damage.

APN is abundant in the blood circulation and has various functions, such as improving insulin resistance [16], antiatherosclerosis [17], regulating microcirculation [18], reducing endothelial cell apoptosis, and antiarterial thrombosis. In addition, it is worth noting that APN can inhibit the apoptosis of cells by regulating the expression of Bax, Bcl-2, and cleaved caspase-3 [19]. At the same time, studies in vitro have found that APN can regulate the proliferation and apoptosis of SMCs by regulating mitochondrial fusion protein 2 (MFN2) and ERK1/2 signaling pathway [20]. Furthermore, APN abrogated tumor necrosis factor- (TNF-) *α*-activated plasminogen activator inhibitor- (PAI-) 1 expression by activating cAMP-PKA-AMPK-NF-*κ*B signaling in human umbilical vein endothelial cells (HUVECs) [21]. Other than that, APN inhibited inflammatory response of microglia to amyloid-*β* oligomer (A*β*O) via AdipoR1-AMPK-NF-*κ*B signaling [22], and APN inhibited palmitate-induced apoptosis by suppression of ROS generation via both the cAMP/PKA and AMPK pathways in HUVECs [23].

In recent years, although there are many reasons for causing apoptosis, it is undeniable that the formation of oxidative stress caused by hypoxia and the large accumulation of ROS are one of the reasons [24]. It is well known that among various molecules related to oxidative stress, SOD is an important factor existing in mammals and humans, which plays an irreplaceable role in regulating oxidative stress damage [25]. It is worth noting that CoCl_2_ can produce oxidative stress, induce cell damage, reduce cell mitochondrial membrane potential, activate caspase family, and ultimately induce apoptosis. Based on this biological characteristic, CoCl_2_ can be used for hypoxic preconditioning in many cell types [26]. We found that APN pretreated could increase SOD activity and decrease MDA levels which is affected by CoCl_2_. Thus, the antioxidant and antiapoptotic effects of APN were probably achieved through reduced formation of oxygen free radicals and regulation of SOD activity and MDA levels, which enhanced the ability of cells to resist oxidative damage.

cAMP is an important “second messenger” in cells, and it is important in intracellular signaling and affects various cellular functions. In addition, cAMP plays a key role in the G protein-mediated signaling pathway. When stimulated extracellularly, cAMP rapidly multiplies in a short period of time, forming an intracellular signal [27]. Another research has found that activation of endogenous WAT Ucn2/3 autocrine/paracrine pathway was involved in hypoxia-induced lipolysis via CRHR2-cAMP-PKA signaling pathway in a hypoxic model [28]. In our study, we found that CoCl_2_ could significantly reduce the level of cAMP and the activity of PKA in SMCs, whereas APN could block the above effects. But how does APN activate the cAMP/PKA signaling pathway? Probably by activating AMPK pathway and its receptors AdipoR1 and AdipoR2; this issue needs further exploration. In addition, what deserves our attention is that the location of the protein on the cell determines its biological function and physiological significance [29]. Moreover, our immunofluorescence results showed that PKA was mainly distributed in the cytoplasm, and the Bax was mainly distributed in the cytoplasm, while Bcl-2 and caspase-3 were mainly distributed in the nucleus. Our results show that PKA plays its biological role mainly in the cytoplasm, while caspase-3 plays its role in the nucleus.

Studies have reported that at least three pathways, namely, cell surface death receptor pathway, intracellular mitochondrial pathway, and Ca^2+^-mediated endoplasmic reticulum pathway, are involved in the occurrence and development of apoptosis [30]. The Bcl-2 family located on the mitochondrial membrane plays a key role in regulating the development of apoptosis, which can be classified into antiapoptotic proteins and proapoptotic proteins according to action [31]. Studies have shown that the expression of Bax on the mitochondrial membrane is enhanced, and a large number of homodimers Bax/Bax can be formed to increase apoptosis, whereas cells are stimulated by harmful substances. In contrast, when Bcl-2 is overexpressed, Bcl-2/Bax heterodimers are formed in large amounts and against the apoptosis of cells; thus, the ratio of Bax and Bcl-2 expression determines apoptosis or survival [32]. Moreover, studies have shown that activating caspase-9 can further activate caspase-3, which is the most important apoptotic performer in the caspase family [33]. Furthermore, we found that, after treatment with CoCl_2_, the apoptosis rate of SMCs was significantly increased, whereas the ratio of Bcl-2/Bax was significantly decreased, the expression of activated caspase-3 was increased, and APN reversed the above changes.

In the present study, APN protected the SMCs of the SMA from CoCl_2_-induced injury not only by adjusting the SOD activity and MDA levels but also by regulating the expression levels of Bcl-2, Bax, and cleaved caspase-3 via activating cAMP/PKA signaling pathway. Our results reveal a link between APN and SMCs apoptosis, suggesting that APN may be a promising treatment for diseases related to circulatory disturbances in the inner ear.

## 5. Conclusion

In summary, our study demonstrates that APN protects SMCs of the SMA from CoCl_2_-induced injury via cAMP/PKA signaling pathway in cells. Therefore, APN might be a promising treatment for diseases related to circulatory disturbances in the inner ear.

## Data Availability

All data generated or analyzed during this study are included in this article.

## References

[1] Attanasio G., Cagnoni L., Masci E. (2017). Chronic cerebrospinal venous insufficiency as a cause of inner ear diseases. *Acta Oto-Laryngologica*.

[2] Kurtaran H., Acar B., Ocak E., Mirici E. (2016). The relationship between senile hearing loss and vestibular activity. *Brazilian Journal of Otorhinolaryngology*.

[3] Krishnamoorthy G., Reimann K., Wangemann P. (2016). Ryanodine-induced vasoconstriction of the gerbil spiral modiolar artery depends on the Ca^2+^ sensitivity but not on Ca^2+^ sparks or BK channels. *BMC Physiology*.

[4] Minakata T., Inagaki A., Yamamura A., Yamamura H., Sekiya S., Murakami S. (2019). Calcium-sensing receptor is functionally expressed in the cochlear perilymphatic compartment and essential for hearing. *Frontiers in Molecular Neuroscience*.

[5] Wangemann P., Wonneberger K. (2005). Neurogenic regulation of cochlear blood flow occurs along the basilar artery, the anterior inferior cerebellar artery and at branch points of the spiral modiolar artery. *Hearing Research*.

[6] Miwa T., Ohta K., Ito N. (2020). Tsukushi is essential for the development of the inner ear. *Molecular Brain*.

[7] Chen Y., Zhao Q., Yang X., Yu X., Yu D., Zhao W. (2019). Effects of cobalt chloride on the stem cell marker expression and osteogenic differentiation of stem cells from human exfoliated deciduous teeth. *Cell Stress & Chaperones*.

[8] Kaymakci M., Acar M., Burukoglu D. (2015). The potential protective effects of 2-aminoethyl diphenylborinate against inner ear acoustic trauma: experimental study using transmission and scanning electron microscopy. *The Journal of International Advanced Otology*.

[9] Li H., Yang S., Wu J. (2018). cAMP/PKA signaling pathway contributes to neuronal apoptosis via regulating IDE expression in a mixed model of type 2 diabetes and Alzheimer's disease. *Journal of Cellular Biochemistry*.

[10] Wang X., Buechler N., Yoza B., McCall C., Vachharajani V. (2016). Adiponectin treatment attenuates inflammatory response during early sepsis in obese mice. *Journal of Inflammation Research*.

[11] Han X., Wu Y., Liu X. (2017). Adiponectin improves coronary no-reflow injury by protecting the endothelium in rats with type 2 diabetes mellitus. *Bioscience Reports*.

[12] Xiao J., Zhang Z., Zhang W. (2018). Primary cultivation and identification of vascular smooth muscle cells from the spiral modiolar artery of guinea pigs. *Medical Science Monitor*.

[13] Yoo S. Y., Yoo J. Y., Kim H. B., Baik T. K., Lee J. H., Woo R. S. (2019). Neuregulin-1 protects neuronal cells against damage due to CoCl2-induced hypoxia by suppressing hypoxia-inducible factor-1*α* and P53 in SH-SY5Y cells. *International Neurourology Journal*.

[14] Zhao X., Liu L., Li R. (2018). Hypoxia-inducible factor 1-*α* (HIF-1*α*) induces apoptosis of human uterosacral ligament fibroblasts through the death receptor and mitochondrial pathways. *Medical Science Monitor*.

[15] Kurata N., Schachern P. A., Paparella M. M., Cureoglu S. (2016). Histopathologic evaluation of vascular findings in the cochlea in patients with presbycusis. *JAMA Otolaryngology. Head & Neck Surgery*.

[16] Ahlstrom P., Rai E., Chakma S., Cho H. H., Rengasamy P., Sweeney G. (2017). Adiponectin improves insulin sensitivity via activation of autophagic flux. *Journal of Molecular Endocrinology*.

[17] Wang X., Chen Q., Pu H. (2016). Adiponectin improves NF-*κ*B-mediated inflammation and abates atherosclerosis progression in apolipoprotein E-deficient mice. *Lipids in Health and Disease*.

[18] Tsuda K. (2011). Roles of adiponectin and oxidative stress in the regulation of membrane microviscosity of red blood cells in hypertensive men-an electron spin resonance study. *Journal of Obesity*.

[19] Shibata R., Sato K., Kumada M. (2007). Adiponectin accumulates in myocardial tissue that has been damaged by ischemia-reperfusion injury via leakage from the vascular compartment. *Cardiovascular Research*.

[20] Wei C., Li Y., Zheng H. (2012). Globular adiponectin protects H9c2 cells from palmitate-induced apoptosis via Akt and ERK1/2 signaling pathways. *Lipids in Health and Disease*.

[21] Chen Y., Zheng Y., Liu L. (2017). Adiponectin inhibits TNF-*α*-Activated PAI-1 expression via the cAMP-PKA-AMPK-NF-*κ*B axis in human umbilical vein endothelial cells. *Cellular Physiology and Biochemistry*.

[22] Jian M., Kwan J. S.-C., Bunting M., Ng R. C.-L., Chan K. H. (2019). Adiponectin suppresses amyloid-*β* oligomer (A*β*O)-induced inflammatory response of microglia via AdipoR1-AMPK-NF-*κ*B signaling pathway. *Journal of Neuroinflammation*.

[23] Kim J. E., Song S. E., Kim Y. W. (2010). Adiponectin inhibits palmitate-induced apoptosis through suppression of reactive oxygen species in endothelial cells: involvement of cAMP/protein kinase A and AMP-activated protein kinase. *The Journal of Endocrinology*.

[24] Kang Y. W., Kim Y. S., Park J. Y. (2020). Hypoxia-induced apoptosis of astrocytes is mediated by reduction of dicer and activation of caspase-1. *Cell Biology International*.

[25] Liu B., Wei H., Lan M., Jia N., Liu J., Zhang M. (2020). MicroRNA-21 mediates the protective effects of salidroside against hypoxia/reoxygenation-induced myocardial oxidative stress and inflammatory response. *Experimental and Therapeutic Medicine*.

[26] Tripathi V. K., Subramaniyan S. A., Hwang I. (2019). Molecular and cellular response of co-cultured cells toward cobalt chloride (CoCl2)-induced hypoxia. *ACS Omega*.

[27] Loberg R. D., Vesely E., Brosius F. C. (2002). Enhanced glycogen synthase kinase-3*β* activity mediates hypoxia-induced apoptosis of vascular smooth muscle cells and is prevented by glucose transport and metabolism. *Journal of Biological Chemistry*.

[28] Xiong Y., Qu Z., Chen N. (2014). The local corticotropin-releasing hormone receptor 2 signalling pathway partly mediates hypoxia-induced increases in lipolysis via the cAMP-protein kinase A signalling pathway in white adipose tissue. *Molecular and Cellular Endocrinology*.

[29] Cheng L., Fan K., Huang Y., Wang D., Leung K. S. (2017). Full characterization of localization diversity in the human protein interactome. *Journal of Proteome Research*.

[30] Liu C., Zhang K., Shen H., Yao X., Sun Q., Chen G. (2018). Necroptosis: a novel manner of cell death, associated with stroke (review). *International Journal of Molecular Medicine*.

[31] Pei X., Li Y., Zhu L., Zhou Z. (2020). Astrocyte-derived exosomes transfer miR-190b to inhibit oxygen and glucose deprivation-induced autophagy and neuronal apoptosis. *Cell Cycle*.

[32] Maes M. E., Schlamp C. L., Nickells R. W. (2017). BAX to basics: how the BCL2 gene family controls the death of retinal ganglion cells. *Progress in Retinal and Eye Research*.

[33] Zhang J., Xia Y., Xu Z., Deng X. (2016). Propofol suppressed hypoxia/reoxygenation-induced apoptosis in HBVSMC by regulation of the expression of Bcl-2, Bax, caspase3, Kir6.1, and p-JNK. *Oxidative Medicine and Cellular Longevity*.

